# Estimating vehicle carbon dioxide emissions from Boulder, Colorado, using horizontal path-integrated column measurements

**DOI:** 10.5194/acp-19-4177-2019

**Published:** 2019

**Authors:** Eleanor M. Waxman, Kevin C. Cossel, Fabrizio Giorgetta, Gar-Wing Truong, William C. Swann, Ian Coddington, Nathan R. Newbury

**Affiliations:** 1 Applied Physics Division, Physical Measurement Laboratory, National Institute of Standards and Technology, 325 Broadway, Boulder, CO 80305, USA; a now at: Crystalline Mirror Solutions LLC, Santa Barbara, CA 93101, USA

## Abstract

We performed 7.5 weeks of path-integrated concentration measurements of CO_2_, CH_4_, H_2_O, and HDO over the city of Boulder, Colorado. An open-path dual-comb spectrometer simultaneously measured time-resolved data across a reference path, located near the mountains to the west of the city, and across an over-city path that intersected two-thirds of the city, including two major commuter arteries. By comparing the measured concentrations over the two paths when the wind is primarily out of the west, we observe daytime CO_2_ enhancements over the city. Given the warm weather and the measurement footprint, the dominant contribution to the CO_2_ enhancement is from city vehicle traffic. We use a Gaussian plume model combined with reported city traffic patterns to estimate city emissions of on-road CO_2_ as (6.2 ± 2.2) × 10^5^ metric tons (t) CO_2_ yr^−1^ after correcting for non-traffic sources. Within the uncertainty, this value agrees with the city’s bottom-up greenhouse gas inventory for the on-road vehicle sector of 4.5 × 10^5^ t CO_2_ yr^−1^. Finally, we discuss experimental modifications that could lead to improved estimates from our path-integrated measurements.

## 1 Introduction

Measurements of greenhouse gases, especially CO_2_ and CH_4_, are critical for monitoring, verification, and reporting as countries and cities work towards decreasing their carbon emissions. Measurements on the city-scale are critical because cities contribute a large fraction of global emissions (Marcotullio et al., 2013; Seto et al., 2014). However, quantification of city greenhouse gas emissions is challenging, especially for CO_2_ since it has a high background and numerous point and diffuse sources including traffic, power plants, and animal and plant respiration. Emissions of pollutants are typically determined using two methods: (1) a top-down approach using atmospheric measurements over a specific site or area to adjust a prior model and (2) bottom-up inventories that calculate emissions based on sector activity and sector emissions factors. Here we demonstrate a technique for top-down measurements that uses an open-path sensor rather than a point sensor and apply it to measure city-scale CO_2_ emissions.

Quantification of CO_2_ fluxes from cities has been determined from eddy covariance flux measurements with a point sensor located on a tower in or near a city (Nemitz et al., 2002; Velasco et al., 2005, 2014; Coutts et al., 2007; Bergeron and Strachan, 2011). However, for a single sensor, the relatively small footprint of the eddy covariance flux measurements limits the utility of this technique for large cities, as do violations of the horizontal homogeneity assumption (Järvi et al., 2018). To overcome this limitation, tower networks of point sensors can measure CO_2_ at multiple sites within a city and at background sites outside the city (McKain et al., 2012; Lauvaux et al., 2013, 2016; Bréon et al., 2015; Shusterman et al., 2016; Staufer et al., 2016; Mueller et al., 2017; Verhulst et al., 2017; Mitchell et al., 2018; Sargent et al., 2018). To distinguish the small enhancements compared to the large background, these networks often use expensive, high-precision cavity ring-down (CRDS) instruments, resulting in a high cost. The BEACO_2_N network (Shusterman et al., 2016), on the other hand, has a much lower cost per sensor. It requires calibration for quantitative results, but the high density of the point sensors can provide lower sensitivity to systematics (Turner et al., 2016). All of these methods use an inversion to determine the total emissions and thus rely on well-known priors and high-resolution mesoscale atmospheric models.

More recently, several other approaches have also been applied to city-scale emissions. Aircraft mass balance measurements (White et al., 1976; Ryerson et al., 2001) have been used to determine city emissions (Mays et al., 2009; Heimburger et al., 2017). However, the use of an aircraft is costly and labor intensive and, therefore, not suited to long-term continuous measurements. Column measurements from the Total Carbon Column Observation Network (TCCON) were used to calculate total South Coast Air Basin (SoCAB) CO and CH_4_ emissions but not CO_2_ (Wunch et al., 2009). In addition, data from the Orbiting Carbon Observatory satellite (OCO-2) were recently combined with TCCON data to estimate CO_2_ emissions from the Los Angeles (LA) basin (Hedelius et al., 2018).

As an alternative to these approaches, horizontal, kilometer-scale, open-path instruments could in principle be used to determine CO_2_ emissions from cities. Such instruments are capable of continuous measurements over a large area with a single instrument (e.g., Wong et al., 2016; Dobler et al., 2017; Coburn et al., 2018). These sensors also have the advantage of being insensitive to small changes in local meteorology and are not subject to the same representation errors as point sensors (Ciais et al., 2010). Several such systems have been deployed. A laser absorption spectrometer system (GreenLITE) has mapped CO_2_ concentrations over Paris but has not yet quantified emissions (Dobler et al., 2017). The California Laboratory of Atmospheric Remote Sensing Fourier Transform Spectrometer (CLARS-FTS) is a downward-looking slant column Fourier transform spectrometer (FTS) that scans across 28 measurement targets in the LA Basin to measure CO_2_, CH_4_, and O_2_ (Wong et al., 2015). Based on the measured CH_4_ CO_2_ ratio and the bottom-up CO_2_ inventory from the California Air Resources Board, researchers have calculated the LA Basin CH_4_ emissions (Wong et al., 2016) but not yet the CO_2_ emissions.

Here we present the quantification of city CO_2_ emissions using open-path measurements made with a dual frequency comb spectrometer. While dual-comb spectroscopy is a relatively new technique, it has a unique set of attributes that make it attractive for open path measurements (Rieker et al., 2014; Coddington et al., 2016; Waxman et al., 2017; Coburn et al., 2018). Dual-comb spectroscopy (DCS) is a high-resolution, broadband technique spanning hundreds of wave numbers but with a resolution that exceeds even high-end Fourier transform infrared spectrometers (FTIRs) leading to a negligible instrument lineshape (Coddington et al., 2016). This allows for simultaneous measurements of multiple species and path-integrated temperature with low systematic uncertainty and without the need for instrument calibration. Additionally, the eye-safe, high-brightness, single-transverse-mode output of a frequency comb allows for beam paths exceeding 10 km, while the speed and parallelism of the measurement suppress any spectral distortion from the inevitable turbulence-induced power fluctuations over such a path (Rieker et al., 2014; Waxman et al., 2017).

**Figure 1:**
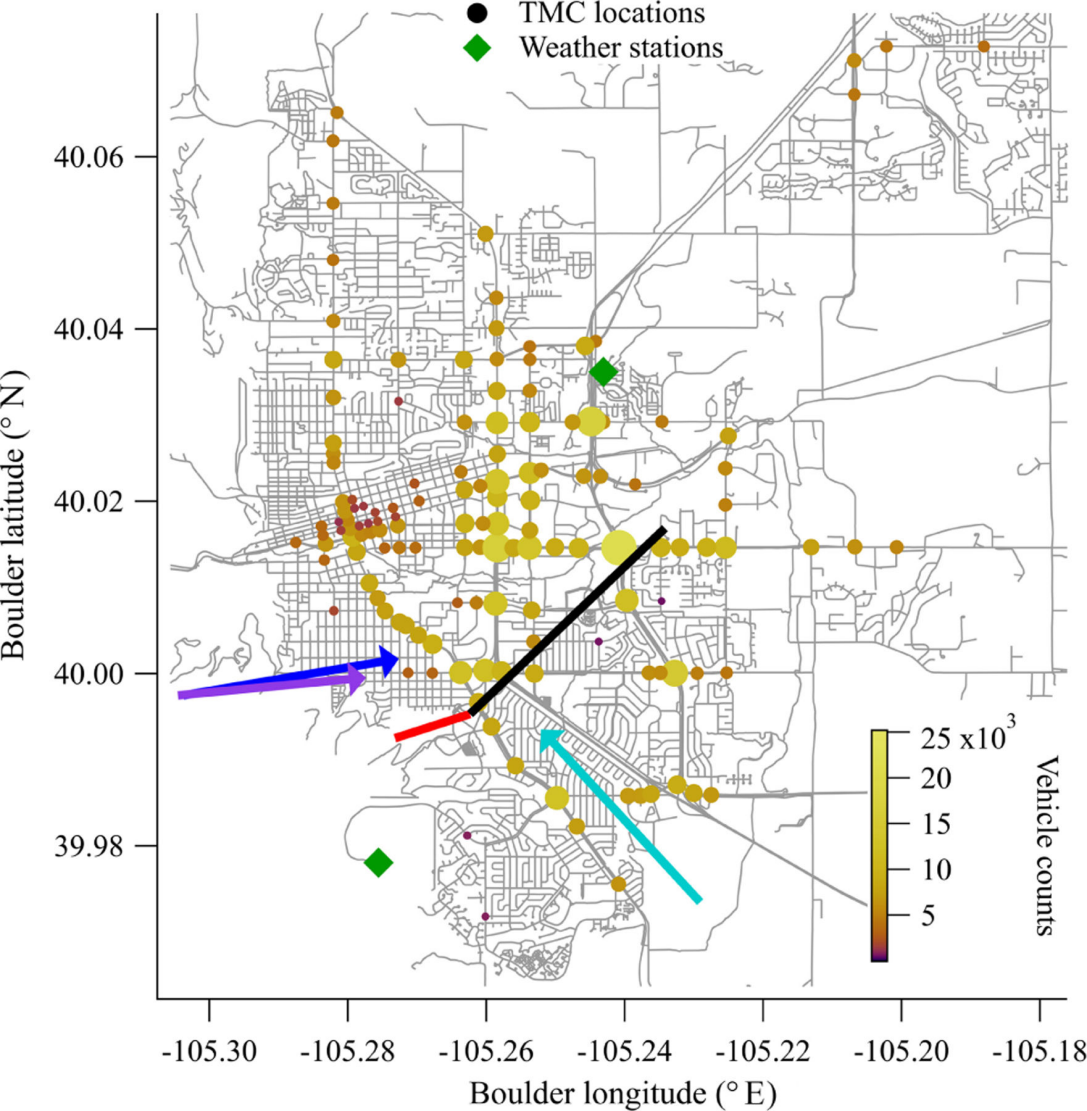
Measurement layout. The two measurement paths are shown by red (reference) and black (over-city) lines. The two weather stations that provided wind speed and direction data are given by the green diamonds. The colored circles are turning movement count (TMC) locations, which are used as a proxy for the traffic source locations. Both color and size represent the number of traffic counts at each location. Dominant wind directions for the campaign overall (aqua) and the test case days (purple for 22 October and blue for 25 October) are given by colored arrows.

Figure 1 shows the measurement layout for an initial campaign to quantify CO_2_ emissions from Boulder, Colorado. Here we take the light from a dual comb spectrometer near the edge of the city and simultaneously measure two paths: a reference path that points west-southwest towards the mountains and an over-city path that crosses the city to the northeast, covering the main traffic arteries of the city with sensitivity to traffic emissions. We acquire time-resolved data at 5 min resolution of CO_2_, CH_4_, H_2_O and isotopologues over 7.5 weeks. The dry mole fraction of CO_2_ shows a diurnal cycle consistent with a morning build-up from traffic followed by a midday decline due to the rising boundary layer. In addition, there is a distinct difference between the weekday and weekend cycles for CO_2_, consistent with traffic patterns. In order to demonstrate the utility of this method for emissions quantification, we perform a preliminary estimate of the CO_2_ emissions from traffic. To do this, we filter the data for days when the wind is out of the west and not too strong so that there is a measurable daytime enhancement in CO_2_ between the reference path and over-city path. Given the weather, beam path location, and observation times, the dominant contribution will be from traffic rather than residential or industrial emissions. We apply a Gaussian plume model to calculate the city emissions based on the expected distributed source (due to traffic) and the path-averaged concentrations. After adjusting for small expected contributions from residential sources and a local utility plant, the measured emission value is scaled to annual city-wide emissions based on city traffic count data. We estimate (6.2 ± 2.2) × 10^5^ metric tons (t) CO_2_ yr^−1^, compared to the bottom-up City of Boulder inventory estimate of 4.46 × 10^5^ t CO_2_ yr^−1^. Finally, we discuss improvements to this estimate, which could be realized by more advantageous beam paths that sample a larger spatial and temporal fraction of the full city emissions and by a more detailed inventory model.

## 2 Experimental data

### 2.1 DCS measurements

**Figure 2:**
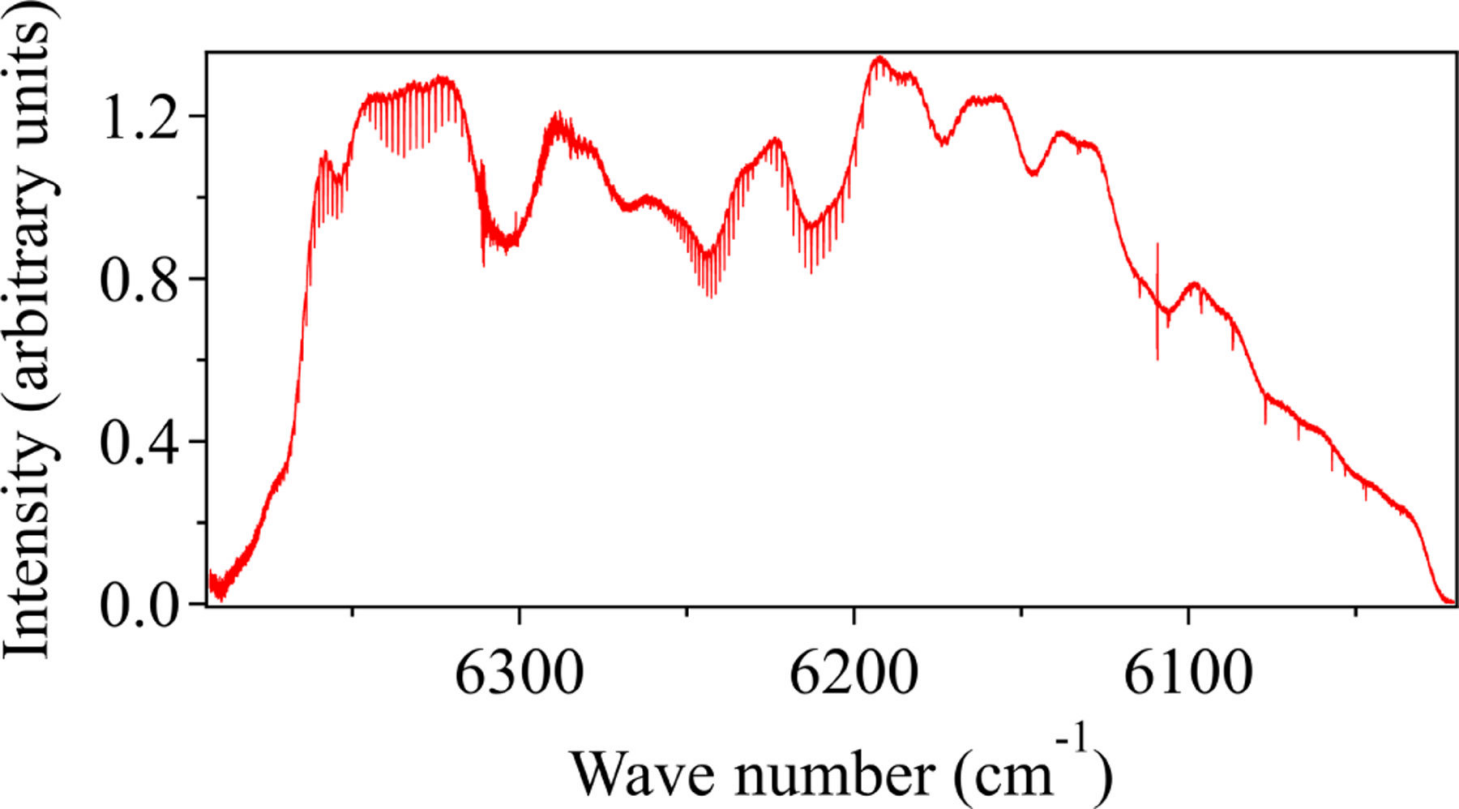
Typical 32 s spectrum measured over the 2 km reference path. CO_2_ bands are observed in the 6350 and 6225 cm^−1^ regions, while CH_4_ and H_2_O are measured between 6150 and 6050 cm^−1^. The larger, slowly varying structure is from the comb intensity profile. The atmospheric absorption appears as the small and narrow dips.

The DCS system was located on the top floor of the National Institute of Standards and Technology (NIST) building in Boulder, Colorado. This instrument has been described previously (Truong et al., 2016; Waxman et al., 2017). The light from the combs is split to generate two combined dual-comb outputs, one of which is transmitted over the reference path and one of which is transmitted over the city path (see Fig. 1). Here, we transmit 2–10 mW of light spanning 1.561 to 1.656 µm (6410 to 6040 cm^−1^), which includes absorption lines from CO_2_, CH_4_, H_2_O and HDO. The returning light from each path is detected and digitized to yield the transmitted optical spectrum at a point spacing of 0.0067 cm^−1^ (1.5 pm) and with effectively perfect (10 ppb) frequency accuracy and narrow instrument lineshape (~ 4 × 10^−6^ cm^−1^). A typical spectrum from the reference path is shown in Fig. 2. A fit of this transmitted spectrum yields the path-averaged gas concentrations. The absolute frequency accuracy and high frequency resolution of the dual-comb spectrometers translates to high precision and accuracy in the retrieved concentrations. Further, DCS spectra are undistorted by turbulence due to the simultaneous acquisition of all spectral channels and the fast sample rate of the instrument (1.6 ms spectrum^−1^, averaged up to 5 min here) (Rieker et al., 2014).

In previous work (Waxman et al., 2017), we confirmed the high precision and accuracy possible with open-path DCS. Two DCS instruments, constructed by different teams, measured atmospheric air over adjacent paths over a 2-week period. The retrieved path-averaged gas concentrations agreed to better than 0.6 ppm (0.14 %) for CO_2_ and 7 ppb (0.35 %) for CH_4_ across the full 2-week period, where the analysis of the two DCS instruments used a common spectral database (HITRAN 2008, Rothman et al., 2009) to retrieve the concentrations from the absorption spectrum. In the work here, a single DCS instrument probes the concentrations across two different open paths simultaneously, which should further suppress any systematic offsets to below 0.45 ppm (Waxman et al., 2017). In addition, Waxman et al. (2017) compared the two DCS instruments to a stationary cavity ring-down (CRDS) point sensor whose inlet was approximately at the midpoint of the open path. This comparison actually took place over the reference path during the first 2 weeks of the present work. During that time, we found a roughly constant difference of 3.4 ppm CO_2_ and 17 ppb CH_4_ between the DCS and CRDS systems. At present, we attribute this offset to differences in the calibration scheme as the DCS is tied to the HITRAN database while the CRDS is tied to the manometric (or gravimetric depending on the gas) World Meteorological Organization (WMO) scale. Similar level offsets have been observed in comparison with the TCCON open-path FTS instrument and point-sensor-based vertical columns resulting in the TCCON CO_2_ scaling factor of 0.9898 (4.08 ppm for a mixing ratio of 400 ppm) (Wunch et al., 2017). This offset does not affect the results here as it is common to both the reference and over-city paths.

The reference and over-city paths had different path lengths and, therefore, used slightly different telescopes and launch powers. For the reference path, 2 mW of dual-comb light is launched from a 5 cm (2 in.) homebuilt off-axis telescope (Cossel et al., 2017; Waxman et al., 2017). The light travels to a 6.35 cm (2.5 in.) retroreflector located on a hilltop 1 km to the southwest of NIST and then is reflected back to a detector that is co-located with the launch telescope for a 1950.17 ± 0.15 m round-trip path. Return powers vary constantly with air turbulence but we collect about 200 µW for a typical 10 dB link loss. For the city path, 10 mW of dual-comb light is launched from a modified 25.4 cm (10 in.) diameter astronomical telescope to a 12.7 cm (5 in.) retroreflector located on a building roof 3.35 km to the northeast for a 6730.66 ± 0.15 m round-trip path. We collect about 100 µW for a typical 20 dB link loss. Round-trip path distances were measured with a laser range finder. Telescope tracking of the retroreflector is implemented to compensate for thermal drifts via a co-aligned 850 nm light-emitting diode (LED) and silicon charge-coupled device camera (Cossel et al., 2017; Waxman et al., 2017).

The measured spectra are analyzed as described in Rieker et al. (2014) and Waxman et al. (2017) at 32 s intervals. Briefly, we fit a seventh-order polynomial and HITRAN data to the measured spectrum in 100 GHz (0.333 cm^−1^) sections to remove the underlying structure from the comb themselves (as opposed to the atmospheric absorption). We fit the resulting absorption spectrum twice: once in the region from 6171 to 6271 cm^−1^ (1.595 to 1.620 µm) to obtain the path-averaged temperature from the 1.6 µm CO_2_ band, and once over the entire spectrum to obtain ^12^CO_2_, ^13^CO_2_, CH_4_, H_2_O, and HDO concentrations using the retrieved temperature. We then use the retrieved H_2_O concentration to correct the wet CO_2_ and CH_4_ mole fractions to dry mole fractions, hereafter referred to as ${\text{X}}_{{\text{CO}}_{2}}$ and ${\text{X}}_{{\text{CH}}_{4}}$, given in units of ppm and ppb (micromole of CO_2_ per mole of dry air, and nanomole of CH_4_ per mole of dry air). The correction equations are ${\text{X}}_{{\text{CO}}_{2}}={\text{CO}}_{2}/\left(1{-\text{H}}_{2}\text{O}\right)$ and ${\text{X}}_{{\text{CH}}_{4}}={\text{CH}}_{4}/\left(1{-\text{H}}_{2}\text{O}\right)$.

**Figure 3:**
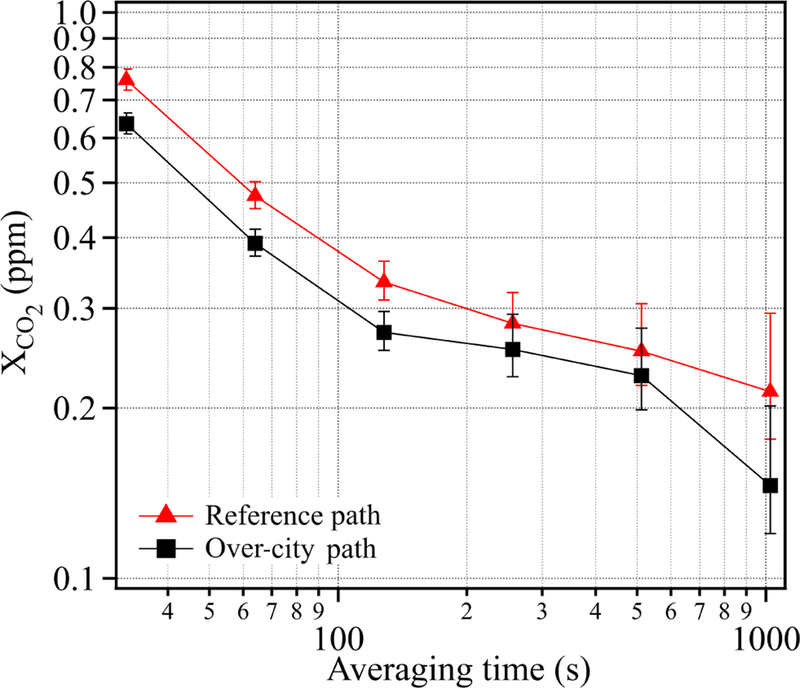
Statistical uncertainty as quantified by the Allan deviations for ${\text{X}}_{{\text{CO}}_{2}}$ over both the reference path (red triangles) and city path (black squares) from a well-mixed 3 h time period on the night of 3 October 2016.

The variations in the retrieved concentrations are due to statistical uncertainty, systematic uncertainty (discussed above), and the true variations in the gas concentrations. Figure 8 of Waxman et al. (2017) quantified the statistical uncertainty in terms of the Allan deviation over the 2 km reference path for both ${\text{X}}_{{\text{CH}}_{4}}$ and ${\text{X}}_{{\text{CO}}_{2}}$. Figure 3 provides an Allan deviation for just ${\text{X}}_{{\text{CO}}_{2}}$ over both the ~ 6.7 km city and ~ 2 km reference paths, as calculated from a relatively “flat” 1000 s period of this measurement campaign on the night of 3 October 2016. As expected, the statistical uncertainty over both paths improves in relation to the square root of integration time until reaching a floor, which we attribute to real variations in the atmospheric gas concentrations. At 30 s, the statistical uncertainty of ${\text{X}}_{{\text{CO}}_{2}}$ is 0.76 ppm for the reference path and 0.64 ppm for the over-city path, finally dropping to 0.21 and 0.15 ppm, respectively, at about 15 min. In most subsequent figures, we show results at a 5 min averaging time for which the statistical uncertainty is well under 0.3 ppm of ${\text{X}}_{{\text{CO}}_{2}}$ for both paths and, therefore, well below the typical atmospheric variations. Note that the uncertainty also improves with path length, as expected due to the stronger absorption. The lower uncertainty over the city path reflects the expected improvement from the 3.4× longer path length lessened by the 2× reduction in return signal power for the longer path length.

### 2.2 Meteorological measurements

Meteorological data including pressure, wind direction, and wind speed are obtained from meteorological stations located at NCAR-Mesa and NCAR-Foothills (ftp://ftp.eol.ucar.edu/pub/archive/weather, last access: 28 March 2019), which are approximately the endpoints of our measurement paths (see Fig. 1), as well as from a 3-D sonic anemometer located at NIST. The path-averaged air temperature was retrieved from the CO_2_ spectra as described above. Finally, we obtain solar insolation from the ATOC weather station located in central Boulder (http://foehn.colorado.edu/weather/atoc1/archive_index.html, last access: 29 March 2019).

### 2.3 Traffic data

We measure a subset of Boulder traffic, so we use the city traffic data to determine the fraction covered by our footprint (see Fig. 1). Traffic data from the City of Boulder are freely available at: https://maps.bouldercolorado.gov/traffic-counts/?_ga=2.264109964.1414067815.1500302174-274759643.1492121882 (last access: 28 March 2019). The city provides two types of traffic data that are useful in this work: the arterial count program (ART) and the turning movement count (TMC) data.

**Figure 4:**
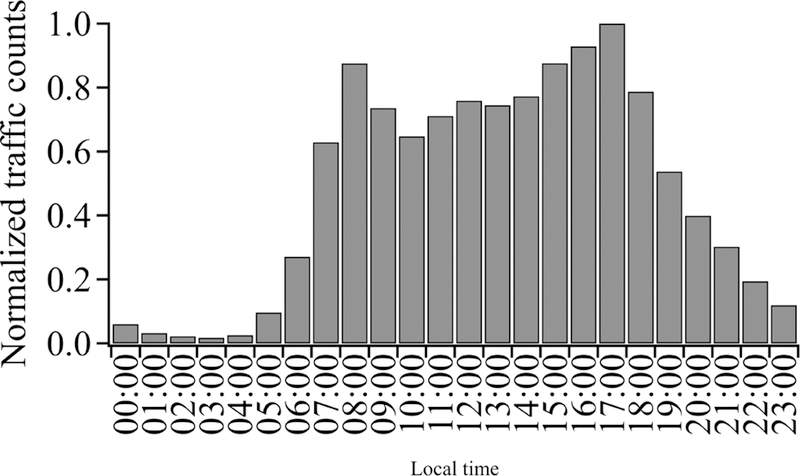
City-wide traffic counts from the Boulder arterial count program (ART), normalized to a peak of unity.

ART measures traffic at 18 major intersections in Boulder for 5 days (1 work week, Monday through Friday) every year in 1 h bins to create a diurnal cycle. The traffic counts for 2016 are shown in Fig. 4. We use these data to scale our selected measurement time periods to a full day as discussed in Sect. 3.3.4. Note that there is only a 10 %–20 % “peak” in traffic counts at the standard commuter times with generally high traffic levels from 07:00 to ~ 19:00, which agrees with the traffic emissions reported by the Hestia inventory model for the similar city of Salt Lake City, UT (Mitchell et al., 2018).

TMC measures the number of vehicles at 140 intersections in Boulder for 1 work day per year during the hours of 07:45–08:45, 12:00–13:00, and 16:45–17:45. One-third of each of these sites is measured every year. We have scaled the 2014 and 2015 data to 2016 traffic levels by using total vehicle mile values available from the City of Boulder. We approximate city vehicle emissions by using the TMC locations as our source locations with a source strength scaled based on the location’s fractional traffic count.

## 3 Results and discussion

### 3.1 DCS measurements

**Figure 5:**
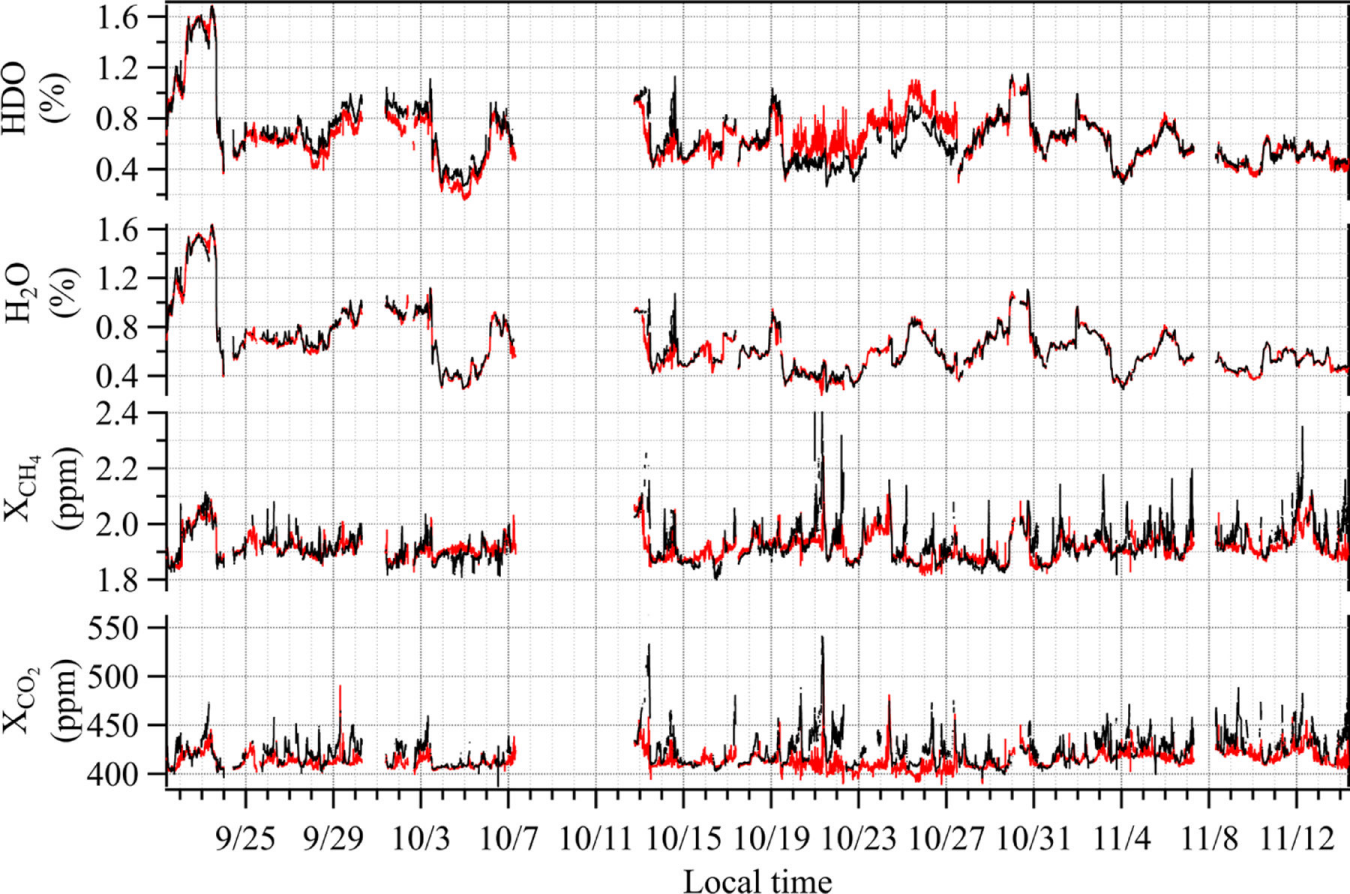
A total of 7.5 weeks of dual-comb spectroscopy data for the reference path (red) and the over-city path (black) smoothed to 5 min time intervals. Enhancements in the over-city path relative to the reference path are observed in CO_2_ and CH_4_ but not in H_2_O or HDO. Note that the HDO concentration includes the HITRAN isotopic scaling.

All 7.5 weeks of DCS measurements of CO_2_, CH_4_, H_2_O, and HDO are shown in Fig. 5. HDO is not used here but is shown for completeness (note that the HDO concentration is scaled by the isotopic abundance in HITRAN). We have insufficient precision to measure time-resolved ^13^CO_2_ concentrations over the 2 km path. However, there are very clear enhancements in the over-city path relative to the reference path for the other trace gases, especially for CO_2_. These enhancements are observed primarily at night when the boundary layer is lower. For example, on 13 October the CO_2_ enhancement reaches 129 ppm and the CH_4_ enhancement reaches 265 ppb. Daytime enhancements occur when the wind speed is very low and intermittent (typically below 5 ms^−1^), which allows emitted gases to build up over the city. When the wind increases to steady, moderate speeds, the concentrations drop quickly as the emissions are flushed out of the city. The H_2_O retrieval is important as accurate knowledge of the time-dependent water concentration is needed to calculate the dry CO_2_ and CH_4_ mole fractions (see Sect. 2.1). Also, the correlation of the water concentration between the two paths indicates the two paths sense the same air mass, which is further substantiated in Fig. 7 and is central to attributing their different CO_2_ concentration to local urban sources.

### 3.2 Diurnal cycles

**Figure 6:**
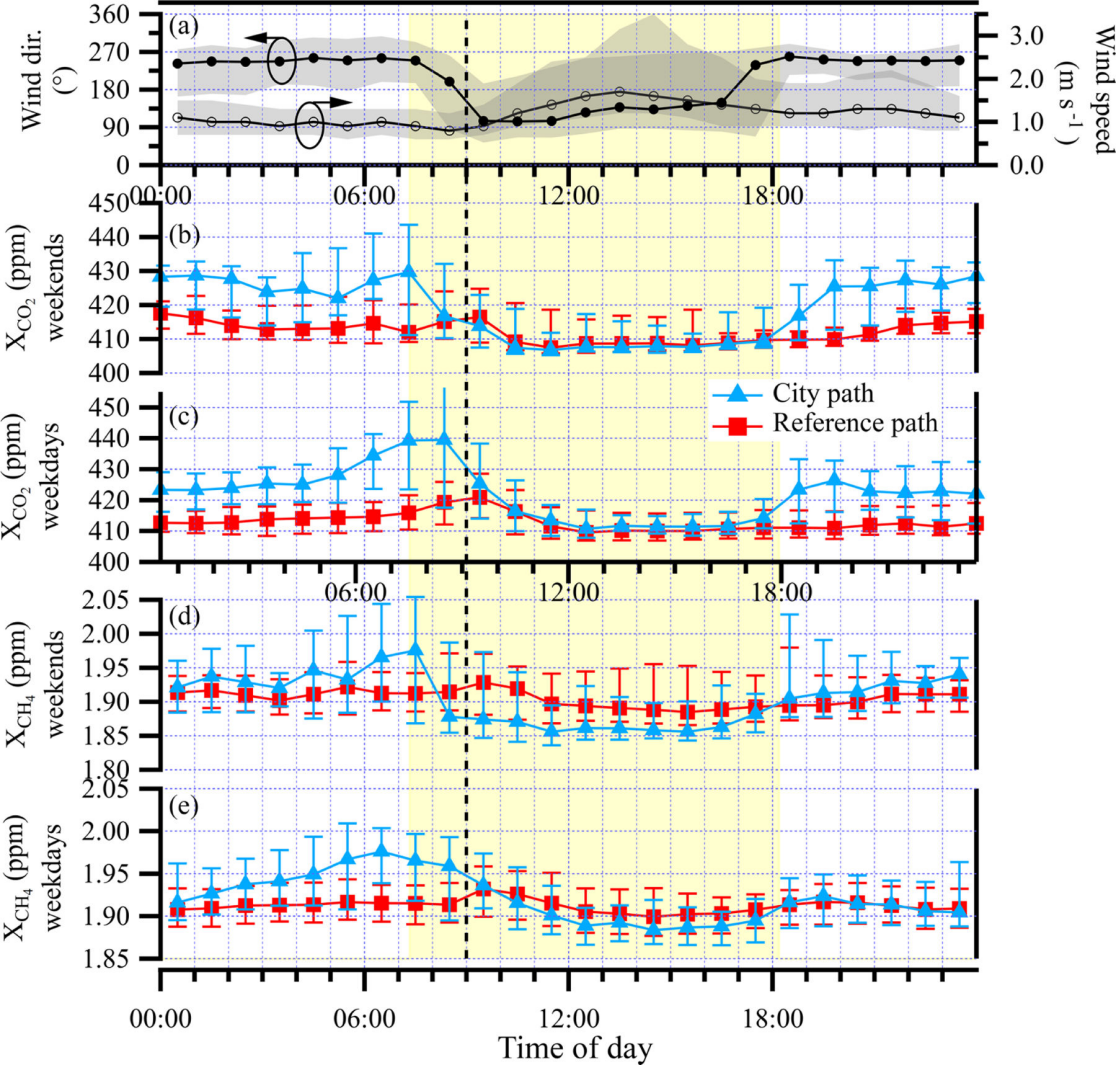
Diurnal cycle analysis. Data are the median of the full 7.5 weeks. **(a)** The mean direction in which the wind is blowing (black trace, left axis) and the wind speed (gray trace, right axis) are both from the NCAR-Foothills measurement station; shaded regions reflect the 25th to 75th quartiles; **(b)** the weekend and **(c)** weekday median ${\text{X}}_{{\text{CO}}_{2}}$ values for the over-city path (blue triangles) and reference path (red squares). Uncertainty bars represent the 25%–75% range of values encountered. Panels **(d)** and **(e)** represent the same data for ${\text{X}}_{{\text{CH}}_{4}}$. The vertical dashed black line marks 09:00 local time and the yellow shaded region highlights the region from sunrise to sunset on 22 October 2016.

The diurnal cycle of ${\text{X}}_{{\text{CO}}_{2}}$ and ${\text{X}}_{{\text{CH}}_{4}}$ for both the reference path and the over-city path are shown in Fig. 6 for weekdays (midnight to midnight Monday through Friday) and weekends (midnight to midnight Saturday and Sunday). We choose to include Monday as a weekday and Saturday as a weekend because the influence of emissions from the previous day is expected to be low. The diurnal cycles of the wind direction and the wind speed measured at NCAR-Foothills are also shown in the top panel of Fig. 6. All diurnal cycles are the median values over the full 7.5 weeks of measurements and the bars reflect the 25 %/75 % quartile values.

The diurnal cycle of the reference path CO_2_ is nearly flat and nearly identical for both weekends and weekdays. It has a slight maximum between 09:00 and 10:00, with average values of 410 to 420 ppm. The diurnal cycle of the city path CO_2_ shows a different trend with a stronger diurnal variation. Overnight from about 18:00 to 09:00, there is an enhancement in the CO_2_ relative to the reference path as the CO_2_ from the city sources builds up due to the low winds out of the west and a presumed collapsing nighttime boundary layer. During the weekdays, this enhancement increases in the morning consistent with the rise in traffic. After the morning, the combination of the presumed rising boundary layer, increased wind speed, and shift in average wind direction out of the west (270°) to the southeast (135°) result in a drop in the city path CO_2_. Moreover, this shift in wind direction means that the reference path no longer samples the clean air from the direction of the mountains but rather sees a very similar CO_2_ enhancement to the city path. Fortunately, as discussed below, there are days when the wind does not shift direction, so there is a measured enhancement of the city path compared to the reference path. In the early evening, as the wind speed drops and the wind direction shifts back to out of the west, the enhancement of the city path over the reference path reappears and continues overnight as the boundary layer presumably drops. In general, the CO_2_ mixing ratios tend to be higher on the weekdays, sometimes exceeding 500 ppm, while weekend mixing ratios are entirely below 490 ppm. This difference is reflected in the median values as well, which reach about 440 ppm during the weekdays but only 430 ppm during the weekend.

The diurnal cycle of the reference path CH_4_ is relatively flat for both weekends and weekdays at just over 1.9 ppm, with a slight peak between 09:00 and 10:00. The diurnal cycle of the city path CH_4_ shows an enhancement, relative to the reference path, between midnight and about 09:00. We attribute this enhancement to sources of CH_4_ within the city, combined again with low nighttime winds and collapsing boundary layer. These sources may be leaking natural gas infrastructure such as observed in Boston (Phillips et al., 2013; McKain et al., 2015; Hendrick et al., 2016), Washington, D.C. (Jackson et al., 2014), and Indianapolis (Lamb et al., 2016). Unlike for CO_2_, the CH_4_ diurnal cycle appears unrelated to traffic (nor would we expect it to be for clean-burning vehicles) as it does not increase during high traffic times.

### 3.3 Estimate for CO_2_ emissions due to traffic

#### 3.3.1 Measurement day selections

**Figure 7:**
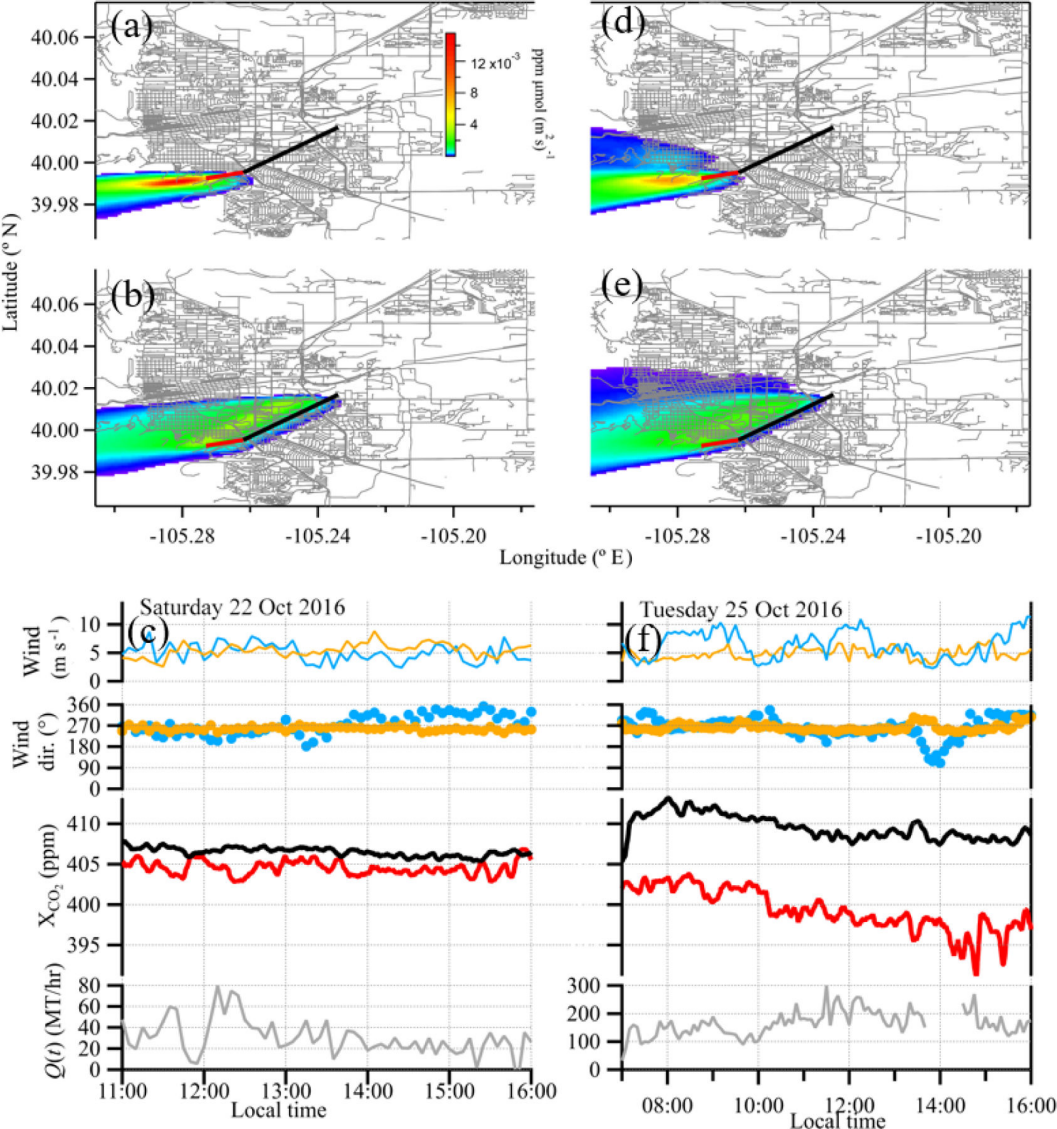
Footprint calculations and time series data for the 2 case study days. Left column – Saturday, 22 October 2016 data; right column – Tuesday, 25 October 2016 data. Upper panels **(a, d)**: footprints for the reference path. Middle panels **(b, e)**: footprints for the over-city path. The footprints are averaged over the respective time windows and open paths. Lower panels **(c, f)**: wind and CO_2_ data at 5 min time intervals. Reference and over-city measurement paths are shown in red and black, respectively. Data plots show ${\text{X}}_{{\text{CO}}_{2}}$ over the reference path (red) and city path (black), wind speed and wind direction measurements taken at NCAR-Mesa (blue) and NCAR-Foothills (orange), and the calculated *Q*(*t*). On 25 October, *Q*(*t*) data near 14:00 have been removed since the reference path wind direction is out of the southeast to the east, resulting in city contamination along the reference path. All data are smoothed to 5 min time intervals.

To select test case days to estimate the city emissions, we filter the ${\text{X}}_{{\text{CO}}_{2}}$ time series for time periods with daytime enhancement and a moderate wind strength predominantly out of the west (270°). Given that the prevailing daytime winds are from the southeast (135°) and often strong, this limits the test case days significantly. However, as is clear from Fig. 1, for these wind conditions, the city path samples a significant fraction of the traffic emissions and the reference path samples no traffic emissions. We only consider daytime enhancements because the nighttime boundary layer behavior is significantly more complicated than a well-mixed daytime stable boundary layer. We find 2 days that meet these criteria: Saturday 22 October 2016 from 11:00 to 16:00 and Tuesday 25 October 2016 from 07:00 to 16:00. Both days have moderate wind speeds (on average, 5 ms^−1^) as measured at both meteorological sites. There are additional days with daytime enhancement in ${\text{X}}_{{\text{CO}}_{2}}$, but the wind direction is variable. Additionally, there are many days with no daytime enhancement in ${\text{X}}_{{\text{CO}}_{2}}$ because the high wind speeds (6 ms^−1^ or higher) prevented buildup of CO_2_. We use 22 October as a proxy for all weekend days and 25 October as a proxy for all weekdays. The ${\text{X}}_{{\text{CO}}_{2}}$ and ${\text{X}}_{{\text{CH}}_{4}}$ mixing ratios, as well as wind speed and wind direction, are shown in Fig. 7 for these 2 case study days.

In order to confirm that the reference path measured clean background air and the over-city path measured city emissions, we calculated footprints for the two test case time periods using the Stochastic Time-Inverted Lagrangian Transport (STILT-R) model (Fasoli et al., 2018). The input meteorology file consisted of a uniform wind field with wind data from the NCAR-Foothills lab, boundary layer height from the North American Regional Reanalysis (NARR), and uniform turbulent velocity variance calculated from the Pasquill stability class (determined from wind speed and solar insolation) from the ground up to the boundary layer. We also used hyper near-field scaling described in Fasoli et al. (2018). Average footprints for the two time periods are shown in Fig. 7. The footprint for the reference path covers undeveloped areas extending from the near foothills into the mountains. The footprint for the over-city path also has contributions from the same general mountain region. In addition, this path has sensitivity to an extended area within the city and, therefore, to a large fraction of the traffic emissions. Note that the open-path geometry leads to a much larger extended footprint for this path than would be the case for a single point sensor located at the same height within the city.

The variability in the reference CO_2_ on both days is a real atmospheric effect. In processing, any data are removed if the signal power is low, which is indicative of poor telescope alignment or strong weather-related attenuation over the beam path, so the variability is not due to variable signal strength. We attribute this variability to the smaller footprint of the reference path relative to the over-city path, as seen in Fig. 7. If the CO_2_ in the air is not fully mixed, then the temporal and spatial variability will be more evident in the path with the smaller footprint.

To convert from the measured enhancement to an emissions rate, we require a model that connects the source strength to the plume concentration. Since we do not have a high-resolution, spatially resolved inventory for Boulder similar to the Hestia model for Salt Lake City (Mitchell et al., 2018), we use the existing Boulder traffic inventory (see Sect. 2.3) in conjunction with a Gaussian plume model.

#### 3.3.2 Gaussian plume calculations

The standard Gaussian plume model that includes total reflection at the Earth’s surface is as follows (Seinfeld and Pandis, 2006):

(1) $$c\left(x,y,z,t\right)=\frac{q}{2\pi \sigma_{y}\sigma_{z}u}\exp\left(\frac{-{\left(y-y_{0}\right)}^{2}}{2\sigma_{y}^{2}}\right)\;\;\;\;\;\;\left[\exp\left(\frac{-{\left(z-H\right)}^{2}}{2\sigma_{z}^{2}}\right)+\exp\left(\frac{-{\left(z+H\right)}^{2}}{2\sigma_{z}^{2}}\right)\right],$$

where (*x*,*y*,*z*) is the location in space for which the plume concentration is being calculated; (*x*_0_*, y*_0_*, H*) is the emissions location; *c*(*x*,*y*,*z*,*t*) is the concentration at location (*x*,*y*,*z*) and time *t*; *q* is the emissions strength (usually in kg s^−1^); *σ*_*y*_ and *σ*_*z*_ are the plume variances in the *y* and *z* direction as a function of travel distance and Pasquill stability class (Seinfeld and Pandis, 2006); and *u* is the wind speed in ms^−1^. The wind is assumed to be in the *x* direction. The plume variances are calculated as follows:

(2) $$\sigma_{y}=\exp\left[I_{y}+J_{y}\left(\ln\Delta x\right)+K_{y}{\left(\ln\Delta x\right)}^{2}\right],$$

and

(3) $$\sigma_{z}=\exp\left[I_{z}+J_{z}\left(\ln\Delta x\right)+K_{z}{\left(\ln\Delta x\right)}^{2}\right],$$

where *I*_*y*_, *J*_*y*_, *K*_*y*_, *I*_*z*_, *J*_*z*_, and *K*_*z*_ are from a look-up table based on the Pasquill stability class, which depends on the wind speed and solar insolation (Seinfeld and Pandis, 2006) and ∆*x* is the *x* distance relative to the plume origin. This plume model does not include any reflection at the boundary layer height; however, due to the small spatial scales, this effect is negligible here.

We modify this equation in several ways: (1) since we measure the column-integrated concentration over a finite beam path at an angle to the wind direction, we integrate the plume concentration along this beam path and then normalize to the length of the beam path; (2) we sum over the emissions locations in the city that contribute emissions to our measurements. Thus, our overall measurement equation is as follows:

(4) $$\left(c-c_{0}\right)=\frac{Q}{L}\sum_{\left(x_{j},y_{j}\right)}\int_{0}^{L}\frac{f_{j}}{2\pi \sigma_{y}\sigma_{z}u}\;\;\;\;\;\;\;\exp\left(\frac{-{\left(s\sin\theta -y_{j}\right)}^{2}}{2\sigma_{y}^{2}}\right)\;\;\;\;\;\;\;\left[\exp\left(\frac{-{\left(15-1\right)}^{2}}{2\sigma_{z}^{2}}\right)+\exp\left(\frac{-{\left(15+1\right)}^{2}}{2\sigma_{z}^{2}}\right)\right]\text{d}s,$$

where (*c*-*c*_0_) is our path-integrated concentration enhancement measurement (in t m^−3^ and t is metric tons; 1 t 1000 kg) along our path *s*, which goes from 0 to *L*; *Q* is the total city emissions in t h^−1^; *L* is our path length in m; (*x*_*j*_, *y*_*j*_) are the source emissions locations; *f*_*j*_ is the fraction of traffic at source location (*x*_*j*_, *y*_*j*_) relative to traffic over all locations in the city from the TMC database; *u* is the wind speed in ms^−1^; *θ* is the angle of the beam path with respect to the wind direction; and *σ*_*y*_ and *σ*_*z*_ are the plume dispersions in m in the *y* and *z* directions, which depend on the sources distance from the beam path. In writing Eq. (4), we assume the wind is in the $+\hat{x}$ direction (this assumption is relaxed below). We assume that all plume emission locations are vehicle tailpipes at 1 m above the ground, and the beam path runs 15 m above ground so all measurement heights are at 15 m above ground.

##### Grid rotation for variable wind directions

To calculate Eq. (4), we grid the emissions locations using UTM (Universal Transverse Mercator) coordinates obtained from Google Earth, where we then define north as $+\hat{y}$ and east as $+\hat{x}$. We translate the coordinate system such that the DCS path begins at the origin (0,0) and travels a distance *L* at angle *θ* with respect to the *x* axis. Eq. (4) is then valid provided the wind is directly in the $+\hat{x}$ direction. More generally, the wind is at a time-varying small angle *φ*(*t*) with respect to $+\hat{x}$. Therefore, we apply a rotation about the origin (Prussin et al., 2015):

$$\left[\begin{array}{ll} \cos\phi & \sin\phi \\ -\sin\phi & \cos\phi \end{array}\right]\left[\begin{array}{l} x\\ y \end{array}\right]=\left[\begin{array}{l} x^{\prime }\\ y^{\prime } \end{array}\right],$$

to generate new traffic coordinates $\left(x_{j}^{'},y_{j}^{'}\right)$ and a new parameterized DCS beam path of (*s* cos(*θ*ʹ), *s* sin(*θ*ʹ)), where *θ*ʹ = *θ* − *φ*(*t*). In this new coordinate system, the wind is along the $+\hat{x}$ direction and Eq. (4) holds with the substitutions *θ* → *θ*ʹ and $y_{j}\to y_{j}^{'}$ and where the *σ*_*y*_ and *σ*_*z*_ are calculated based on the distance $\Delta x=\left|x_{j}^{'}-\left(y_{j}^{'}/\tan\theta^{\prime }\right)\right|$.

##### Time-dependent estimate of *Q*(*t*)

The rotated Eq. (4) can be solved for *Q* in terms of the measured or estimated values of *c*(*t*) − *c*_0_(*t*), *u*(*t*), ∆*φ*(*t*), *σ*_*y*_ (*t*), *σ*_*z*_(*t*), *θ*, *L*, and *f*_*i*_, where the first five quantities are time dependent. The resulting, time-dependent *Q*(*t*) for each test case day is shown in the bottom panels of Fig. 7 and has a mean value and standard deviation of *Q*_22Oct_ = 31 ± 17 t CO_2_ h^−1^ for 22 October and *Q*_25Oct_ = 165 ± 45 t CO_2_ h^−1^ for 25 October for the 5 min averaged data as shown.

##### Uncertainty in *Q*(*t*)

Seven measured parameters factor into the emissions calculation of *Q*(*t*) for the 2 days. These are given in Table 1 along with the instrumental measurement precision and the observed variability. Note that solar insolation is used solely in the determination of the Pasquill stability class (Seinfeld and Pandis, 2006). The stability class is relatively insensitive to the variations in solar insolation observed on the 2 test case days. As can be seen in the table, the uncertainty is dominated by the natural variability in parameters like wind speed, wind direction, and CO_2_ concentration rather than the DCS spectrometer precision. The observed variability over the 5–9 h period is typically at least a factor of 2 larger than the instrument precision. The variability in these parameters leads to the observed variability in *Q*(*t*). We use the mean of *Q*(*t*) as our emissions value and the standard deviation (at 5 min time-averaging) as its uncertainty. In using this standard deviation as a measure of the uncertainty, we attempt to capture the uncertainty associated with the discrepancies between, for example, the weather station measurements of wind direction and speed relative to the true wind direction (which results in greater or fewer number of plumes from the given traffic locations intercepting the measurement path). This variability appears in *Q*(*t*) as the nominal measured wind direction varies. Future systems with redundant, distributed DCS beam paths would provide a superior estimate of all of these uncertainties.

In addition, there are assumptions and possible uncertainties inherent to the Gaussian plume model. First, the model does not include the effects of buildings, trees, or other objects that could break up the plume between the emissions location and the beam path. Second, we assume that all CO_2_ emissions come from the discrete locations shown in Fig. 1, while in reality the emissions are likely substantially more diffuse. The assumption of discrete emissions simplifies modeling and is feasible due to the city traffic data but may result in a bias due to the coarse distribution of traffic measurements. Third, we approximate the measurement height at 15 m above ground although the beam height differs over the path since Boulder is not perfectly flat. Finally, we use standard *I*_*y*_, *J*_*y*_, *K*_*y*_, *I*_*z*_, *J*_*z*_, and *K*_*z*_ values that were derived for rural areas (Turner, 1970), which may be different to urban or suburban areas. However, the greatest differences between rural and urban conditions are expected to be at night (Turner, 1970).

Further, we ran plume calculations in STILT-R using both wind fields derived from the local meteorological stations shown in Fig. 1 and using the North American Mesoscale Forecast System (NAM, https://www.ncdc.noaa.gov/data-access/model-data/model-datasets/north-american-mesoscale-forecast-system-nam, last access: 28 March 2019). The High-Resolution Rapid Refresh (HRRR, https://rapidrefresh.noaa.gov/hrrr/, last access: 28 March 2019) and North American Regional Reanalysis (NARR, https://www.ncdc.noaa.gov/data-access/model-data/model-datasets/north-american-regional-reanalysis-narr, last access: 28 March 2019) wind projections did not match the measured winds at the meteorological stations. These calculations produced emissions values ranging between 55 and 770 t h^−1^, depending on the wind fields and vertical dispersion parameterization used. This brackets our emission calculations by approximately a factor of 3 in each direction and shows how sensitive these kilometer-scale measurements are to vertical dispersion.

#### 3.3.3 Corrections for non-traffic sources of CO_2_

There are a number of non-traffic sources of CO_2_ that could contribute to our measured ${\text{X}}_{{\text{CO}}_{2}}$ enhancement, including local power plants, residential emissions, and biological activity. These non-traffic sources should have a relatively minor contribution for several reasons. First, the footprint of the over-city path does not overlap the large power plant to the east of the Boulder city limits. Second, the temperature during the 2 test case days was 24 and 20 °C (68 and 75 °F) on 22 and 25 October, leading to minimal residential and commercial heating. Third, the measurements occurred in October after leaf senescence so there should be negligible biological activity. Nevertheless, as discussed below, we do adjust our measurements to account for the relatively minor contribution from non-traffic sources before scaling up to an estimate of the annual traffic emissions.

We first consider power plants. There are two power generation facilities on the Department of Commerce (DOC) campus located near the NIST building that houses the dualcomb spectrometer: the site’s Central Utilities Plant (CUP), and the National Oceanic and Atmospheric Administration (NOAA) building’s boilers. To calculate their average CO_2_ emissions, we used available fuel consumption data (October 2016 monthly average for the CUP and mid-November to mid-December 2016 average for the NOAA boilers; October data were unavailable) and the EPA emissions factor (EPA, 1995). We then modeled the CUP and boiler plume emissions using WindTrax (Flesch et al., 1995, 2004) with wind speed and direction data from the NCAR-Mesa site. We find that due to the moderate wind speeds (~ 5 ms^−1^) during our case study days and the height mismatch between the emission stacks and our measurement path over the DOC campus, there is negligible enhancement over the reference path. Given the location of the emission sources and the wind direction during our measurement periods, the emissions also do not cross the over-city beam path. Therefore, we apply no correction for these two power plant emissions.

The University of Colorado also has a power plant that falls within the main footprint associated with the over-city beam path, shown in Fig. 7, and whose emissions are expected to intersect our over-city beam path. The EPA Greenhouse Gas Reporting Program (GHGRP, https://www.epa.gov/ghgreporting, last access: 28 March 2019) lists the 2017 emission from the power plant as 2.7 × 10^4^ t CO_2_ or an average of 3.1 t h^−1^ (no breakdown by season or hour is provided). We apply this correction to our previous daily values and add a conservative uncertainty equal to this correction in quadrature with the previous uncertainty. The new adjusted values are then 28 ± 17 t CO_2_ h^−1^ for 22 October and 162 ± 45 t CO_2_ h^−1^ for 25 October.

The large Valmont power station lies just outside the city limits to the east of Boulder; however, given its location and the dominant selected westerly wind, emissions from this source do not reach our beam paths. There are no other power generation facilities within the city that report to the GHGRP, so we make no further corrections based on power plants.

In addition, there are also likely diffuse emissions from residential and commercial furnaces and water heaters that use natural gas. The City of Boulder Community Greenhouse Gas Emissions Inventory reports 20 % of the city emissions, or 3.18 × 10^5^ t CO_2_, were from natural gas in 2016 (https://www-static.bouldercolorado.gov/docs/2016_Greenhouse_Gas_Emissions_Inventory_Report_FINAL-1-201803121328.pdf?_ga=2.130927943.970967930.1525795820–107394975, last access: 28 March 2019). The natural gas usage varies strongly by month with building heating requirements. Although our measurements occurred in October, the measurement days were quite warm (20–24 °C) so that residential and commercial building heating was unlikely and the use of an annual average would overestimate any contribution. Instead, we scale the natural gas usage according to the monthly breakdown provided by the United States Energy Information Administration database for Colorado (https://www.eia.gov/dnav/ng/hist/n3010co2m.htm, last access: 28 March 2019). The mean daytime (approximately sunrise to sunset, 07:00 to 18:00) temperature in October was 18.2 °C while the mean temperature (including day and night) for October was 15.7 °C. Our daytime-only measurements, therefore, had a mean temperature that was much closer to the mean temperature (day and night) of September, which was 19.2 °C. Therefore, we scale the Boulder annual natural gas consumption by the September 2016 natural gas usage, which was 2.4 % of the Colorado annual total (https://www.eia.gov/dnav/ng/hist/n3010co2m.htm, last access: 28 March 2019). The estimated total emissions from residential and commercial natural gas usage in Boulder over our measurement days is then 10.2 t CO_2_ h^−1^. We apply this correction to our measured values and include a (conservative) uncertainty equal to this correction. The new adjusted values are then *Q*_22Oct,adj_ = 18 ± 20 t CO_2_ h^−1^ for 22 October and *Q*_25Oct,adj_ = 152 ± 46 t CO_2_ h^−1^ for 25 October.

Once leaf senescence has completed, neither plants nor soil respiration contribute to CO_2_ signal (Matyssek et al., 2013). The National Phenology Network (USA National Phenology Network, 2018) data show that for the site nearest to Boulder (64 km north of Boulder), the leaf fall dates were 15 September 2016 for box elder trees and 6 October 2016 for the eastern cottonwood. Thus, by our measurement dates leaf senescence should be fully complete and plants will not contribute to the city CO_2_ enhancement. We note that a wide range of biogenic contributions to CO_2_ have been noted in the literature (Gurney et al., 2017; Mitchell et al., 2018; Sargent et al., 2018).

#### 3.3.4 Scaling to annual emissions

In order to compare with the city inventory, we scale our results to an annual total. To do this, we use the hourly traffic data of Fig. 4 to scale *Q*_22Oct,adj_ and *Q*_25Oct,adj_to a daily emission. Based on Fig. 4, 34% of the total traffic counts occur during the 5 h measurement period on 22 October and 52 % of the total traffic counts occur during the 8 h measurement period on 25 October (excluding the 13:00 to 14:00 period). The daily emissions are then *Q*_22Oct,day_ = *Q*_22Oct,adj_ × (5 h)/(0.34) and *Q*_25Oct,day_ = *Q*_25Oct,adj_ × (8 h)/(0.52). The traffic data in Fig. 4 are based on weekday measurement, and we assume that the hourly distribution is the same for weekends; this may lead to a slight overestimate in the weekend data where a larger fraction of emissions occurs between 11:00 and 16:00 than on weekdays. We then scale to annual emissions by assuming that the emissions on 22 October are representative of all 112 weekend and holiday days and the emissions on 25 October are representative of all 253 workdays. Including their uncertainty, this calculation yields (6.2 ± 1.8) × 10^5^ t CO_2_ yr^−1^.

The scaling relies heavily on the traffic count data supplied by the city of Boulder, which do not have an associated uncertainty value. A comparison of these data over several years shows a typical 7 % statistical variation at a given TMC location after removing a linear trend. We assume this reflects day-to-day fluctuations in traffic. In addition, there will be seasonal variations, which are not captured in the extrapolation from our 2 test case days to the annual emissions. Due to the lack of seasonal data for Boulder traffic, we use the detailed Hestia traffic inventory for Salt Lake City, UT, given in Fig. 2 of Mitchell et al. (2018). These data show a variation of ±18 % in traffic emissions between “summer” and “winter” months. Combined in quadrature with the 7 % statistical uncertainty in the TMC traffic count data, this leads to an additional ~ 20 % uncertainty to the scaled annual estimate. As noted earlier, we have not applied any additional uncertainty on the reliance on the TMC data as a proxy for emissions locations.

Including the additional uncertainty on the scaling to annual emissions, we estimate an annual emission rate of (6.2 ± 2.2) × 10^5^ t CO_2_ yr^−1^ for traffic carbon emissions for Boulder, CO.

## 4 Comparison with city estimates

The city vehicle emissions estimate comes from total vehicle miles traveled based on data from the transportation department, miles per gallon inputs from the EPA state inventory tool, and vehicle type distribution from the Colorado Department of Public Health and the Environment (Kimberlee Rankin, City of Boulder, personal communication). The City of Boulder estimates total vehicle emissions of 4.50 × 10^5^ t CO_2_ in 2016 (https://www-static.bouldercolorado.gov/docs/2016_Greenhouse_Gas_Emissions_Inventory_Report_FINAL-1-201803121328.pdf?_ga=2.130927943.970967930.1525795820–107394975, last access: 28 March 2019). On-road emissions account for greater than 99 % of the transportation emissions, so we have scaled this value down by 1 % for an on-road emissions value of 4.46 × 10^5^ t CO_2_. We assume that all traffic emissions are CO_2_ rather than a mix of CO_2_ and CH_4_. There is no uncertainty provided by the city on this value.

In comparison, we estimate (6.2 ± 2.2) 10^5^ t CO_2_ yr^−1^, which is 139 % of the city estimate but agrees within the given uncertainty. Interestingly, other studies have also found that emissions measurements were higher than the reported inventory values. Brioude et al. (2013) found top-down aircraft estimates of Los Angeles county and the South Coast Air Basin CO_2_ were 1.45 times larger than the Vulcan 2005 inventory (Gurney et al., 2009). An earlier aircraft campaign over Sacramento, CA, found an average CO_2_ emission, with 100 % uncertainty, that was 15 %–20 % higher than the Vulcan estimate (Turnbull et al., 2011). Lauvaux et al. (2016) compared Indianapolis city CO_2_ emissions measured by a network of CRDS instruments to the HESTIA inventory (Gurney et al., 2012) during INFLUX (Davis et al., 2017). They found that despite the building-scale resolution in the HESTIA inventory, it still underestimated the annual CO_2_ flux by 20 %. An updated version of HESTIA predicted very similar emissions estimates for on-road, residential, and commercial sectors, so the discrepancy was attributed to missing sources of CO_2_, including animal (primarily human and companion animal) respiration, biofuel combustion, and biosphere respiration (Gurney et al., 2017).

### 4.1 Improvements in future measurements

Future improvements should include additional and different beam paths, selected based on prevailing wind directions. Our initial assumption that the mountain path would generally act as a reference path was incorrect since the prevailing daytime winds during this time of year are not out of the west but rather the southeast. An east–west running beam north of the city and one south of the city would allow us to utilize a larger fraction of the data as the predominant midday wind direction during the fall is out of the north to the northeast (see Fig. 1). Even longer beam paths would also interrogate a larger fraction of the city and measure a correspondingly larger fraction of the vehicle emissions. Vertically resolved data from, e.g., a series of stacked retroreflectors would better test the assumption of vertically dispersing Gaussian plumes.

Additionally, more extensive modeling to cover variable wind directions and speeds would allow the incorporation of a much larger fraction of the data than the 2 days selected here. An inversion-based model similar to Lauvaux et al. (2013) could potentially be applied to a small city like Boulder; however, this would depend heavily on the quality of the bottom-up emissions inventory used to generate the priors. Indeed, one of the major future improvements would be to generate a detailed Hestia inventory of Boulder, CO, similar to that generated for Salt Lake City, UT (Mitchell et al., 2018).

## 5 Conclusions

We demonstrate the use of an open-path dual frequency comb spectroscopy system for quantifying city emissions of carbon dioxide. We send light over two paths: a reference path that samples the concentration of gases entering the city from the west, and an over-city path that measures the concentrations of gases after the air mass has crossed approximately two-thirds of the city, including two major commuter arteries. The measured diurnal cycle shows a significant traffic-related enhancement in the carbon dioxide signal during weekdays in the over-city path compared to the reference path. We select 2 case study days with appropriate wind conditions and apply Gaussian plume modeling to estimate the total vehicular carbon emission. We then scale these results up to annual city-wide emissions using traffic data from the City of Boulder. We find overall traffic-related carbon emissions that are approximately 1.4 times greater than the city’s bottom-up traffic emissions inventory but with an uncertainty that encompasses the city inventory estimate. Further improvements to this method should include improved design of reference and over-city paths and a more detailed inventory model for Boulder CO, which together should further reduce the overall uncertainty in the estimate.

## Figures and Tables

**Table 1.** Parameters used to calculate the emission rate from Eq. (4). The measurement precision refers to the instrument uncertainty in the measurement quantity. The variability refers to the observed environmental variability over the measurement period. The variability from the enhancement, the wind direction, and the wind speed drive the observed variability in the estimated *Q*(*t*). The distance from a given source location to the DCS measurement path, ∆*x*_*j*_, varies with location and has a 5 m uncertainty.

Quantity	Measurement precision	22 October 11:00–16:00		25 October 07:00–16:00	

		Mean	Variability	Mean	Variability
Pathlength *L*	0.15 m	6730.66 m	0	6730.66 m	0
Enhancement (*c-c*_0_)	0.28 ppm (ref.) 0.25 ppm (city)	1.99 ppm	0.97 ppm (49 %)	10.3 ppm	1.9 ppm (19 %)
Wind speed *u*	0.3 ms^−1^	5.2 ms^−1^	1.0 ms^−1^ (19 %)	5.6 ms^−1^	1.3 ms^−1^ (23 %)
Solar insolation	5%	570 W tn^−2^	76 W m^−2^ (13 %)	275 W m^−2^	185 W m^−2^ (67 %)
Wind direction *φ*	2°	265°	21°	264°	15°

## Appendix A: Modification of the Gaussian plume equation

Equation (1) is the standard Gaussian plume equation as discussed in Sect. 3.3.2 (Seinfeld and Pandis, 2006). It is reproduced here:

$$c\left(x,y,z,t\right)=\frac{q}{2\pi \sigma_{y}\sigma_{z}u}\exp\left(\frac{-{\left(y-y_{0}\right)}^{2}}{2\sigma_{y}^{2}}\right)\;\;\;\;\;\;\left[\exp\left(\frac{-{\left(z-H\right)}^{2}}{2\sigma_{z}^{2}}\right)+\exp\left(\frac{-{\left(z+H\right)}^{2}}{2\sigma_{z}^{2}}\right)\right],$$

where the standard variables are as defined in Sect. 3.3.2.

### A1 Path-integrated substitutions

The DCS returns the average concentration along a line path. We denote distance along this path by the variable *s*, where *s* runs from 0 to *L*. This path is assumed to lie in the *x*–*y* plane at an angle *θ* with respect to the *x* axis (which is assumed to be the wind direction in the standard Gaussian plume equation). With these definitions, the contribution to the DCS signal from the plume is as follows:

$$\left(c-c_{0}\right)=\frac{1}{L}\int_{0}^{L}c\left(s\cos\theta ,s\sin\theta ,z,t\right)\text{d}s,$$

or

$$\left(c-c_{0}\right)=\frac{1}{L}\frac{q}{2\pi \sigma_{y}\sigma_{z}u}\int_{0}^{L}\exp\left(\frac{-\left(s\sin\theta -y_{0}\right)}{2\sigma_{y}^{2}}\right)\;\;\;\;\;\;\;\left[\exp\left(\frac{-{\left(z-H\right)}^{2}}{2\sigma_{z}^{2}}\right)+\exp\left(\frac{-{\left(z+H\right)}^{2}}{2\sigma_{z}^{2}}\right)\right]\text{d}s.$$

### A2 Accounting for multiple point sources

Rather than a single source at (*x*_0_, *y*_0_), we have multiple sources at locations (*x*_*j*_, *y*_*j*_), each with a source strength *f*_*j*_*q*, where *f*_*j*_ is the fractional source strength out of the total value *q*. We now sum over all sources to find the total enhancement. We also change the units of *q* from kg s^−1^ to t yr^−1^ and thus change the emissions variable to *Q* to indicate the unit change. This gives the following equation:

$$\left(c-c_{0}\right)=\frac{Q}{L}\sum_{\left(x_{j},y_{j}\right)}\int_{0}^{L}\frac{f_{j}}{2\pi \sigma_{y}\sigma_{z}u}\exp\left(\frac{-{\left(s\sin\theta -y_{j}\right)}^{2}}{2\sigma_{y}^{2}}\right)\;\;\;\;\;\;\;\left[\exp\left(\frac{-{\left(z-H\right)}^{2}}{2\sigma_{z}^{2}}\right)+\exp\left(\frac{-{\left(z+H\right)}^{2}}{2\sigma_{z}^{2}}\right)\right]\text{d}s.$$

### A3 Height substitutions

We assume that the point source emissions locations are 1 m above ground (*z* = 1), and city topographic data indicate that our beam path is approximately 15 m above ground (*H* = 15). These substitutions finally lead to Eq. (4) in the main text.
